# Diagnosis and Incidence of Spondylosis and Cervical Disc Disorders in the University Clinical Hospital in Olsztyn, in Years 2011–2015

**DOI:** 10.1155/2018/5643839

**Published:** 2018-03-25

**Authors:** Małgorzata Kolenkiewicz, Andrzej Włodarczyk, Joanna Wojtkiewicz

**Affiliations:** ^1^Department of Pathophysiology, School of Medicine, Collegium Medicum, University of Warmia and Mazury in Olsztyn, Olsztyn, Poland; ^2^Department of Public Health, Epidemiology and Microbiology, School of Medicine, Collegium Medicum, University of Warmia and Mazury in Olsztyn, University Clinical Hospital in Olsztyn, Olsztyn, Poland

## Abstract

**Background:**

Disorders connected with the musculoskeletal and central nervous system dysfunction are the most significant clinical problem worldwide. Our earlier research has shown that back and spinal disorders and lumbar disc disorders were most frequently diagnosed using MRI scanner at the University Clinical Hospital (UCH) in Olsztyn in years 2011–2015. We have also observed that another two diseases of spinal column, spondylosis and cervical disc disorders, were also very prevalent. The main objective of this work was to analyze the prevalence of spondylosis and cervical disc disorders in the study population diagnosed at UCH in years 2011–2015.

**Methods:**

The digital database including patients' diagnostic and demographic information was generated based on MRI reports from years 2011–2015 and analyzed using SPSS software.

**Results:**

Within the study group (*n* = 13298) the most frequently MRI-diagnosed diseases were musculoskeletal group (M00–M99; *n* = 7711; 57,98%) and cervical disc disorders (M50; *n* = 1659; 12,47%) and spondylosis (M47, *n* = 611; 4,59%). More women (67%) than men (33%) were enrolled in the study, and the largest fraction of the study population was in the range of 51–60 years, with about 1/3 of cases of both diseases diagnosed in early age range of 31–40 years.

**Conclusion:**

Significant number of patients presenting with either of the spine disorders at the young age of 31–40 years points to the necessity of introducing methods preventing disorders of the vertebral column at younger age, preferably at school age.

## 1. Introduction

Spinal disorders become an increasingly important social and medical problem of the modern world. The back pain can result from various pathologies and in 90% of patients, among the main causes are damage and degenerative changes in the intervertebral discs or spondyloarthrosis [1–4]. Our earlier study has shown that in the Warmia and Mazury Province the most common musculoskeletal disorders were different types of back diseases, and among them, intervertebral disc disorders were most prominent ([5]-ibid), which was in agreement with reports from other populations [6–11]. Spondylosis and cervical disc disorders however are also very frequent diseases of spinal column, as was earlier documented [2–4].

Spondyloses occur predominantly in the lumbar part, mainly due to an unfavorable ratio of the mechanical load—usually excessive—to the size of the intervertebral discs [12]. Lumbar spondyloses (LS) are characterized by degenerative changes in the spine, intervertebral disc or facet joints, vertebral body sclerosis, and hypertrophy of spinal column ligaments and others. Moreover, negative functional effects of LS may lead to the loss of spinal mobility [2]. However, spondyloses may occur also in the cervical part of vertebral column and they can be divided into the traumatic and nontraumatic injuries. Traumatic injuries occur mainly during frontal impact, such as a “whiplash” occurring during car accidents [13]. Intervertebral disc herniation and pathological changes in the vertebra as related to age, sex, occupation, and life style belong to the group of nontraumatic injuries [14]. Most of the pathological changes in spondyloses are combination of factors, such as decrease of the disc height or degenerative changes in the joints [15].

Problems in cervical part of spinal column are also connected with neck, arm, and forearm pain, which can significantly decrease the quality of life; this is due to pathological changes impacting cervical spinal nerves forming cervical and brachial plexuses [14]. The neck and shoulder pain are found to be more common disorders than the low back pain [16]; each of them is often connected to specific professions [16–24].

Here we describe the population presenting with spinal and back injuries focusing on spondylosis and cervical disc disorders, diagnosed using MRI at the University* C*linical Hospital in Olsztyn, in years 2011–2015.

## 2. Materials and Methods

The prevalence of spondylosis and cervical disc disorders using the digital database of patients is examined by MRI at the University Clinical Hospital (UCH) in Olsztyn. The database included all MRI scans collected in years 2011–2015, age, sex, and the diagnosis. All disease categories were recorded using an appropriate letter code according to the* International Statistical Classification of Diseases and Related Health Problems,* Tenth Revision (ICD-10) [25]. For further analyses, data of patients presenting with the specific diseases of musculoskeletal system and connective tissue including spondylosis (M47) and cervical disc disorders (M50) were selected and grouped into the given disease subtypes. Sex distribution in each study subtype and in each year of the study was determined, and to identify the age group most frequently diagnosed, study population was divided into ten age groups in the 10-year intervals.

## 3. Results

During five years of the study period, among all spinal diseases diagnosed at the UCH, M47 (spondylosis) and M50 (cervical disc disorders) represented above 4% and 12%, respectively (Table 1). In each of those general groups, specific subtypes were defined, and two biggest subtypes of each group were chosen for detailed analyses.

**Figure 1:**
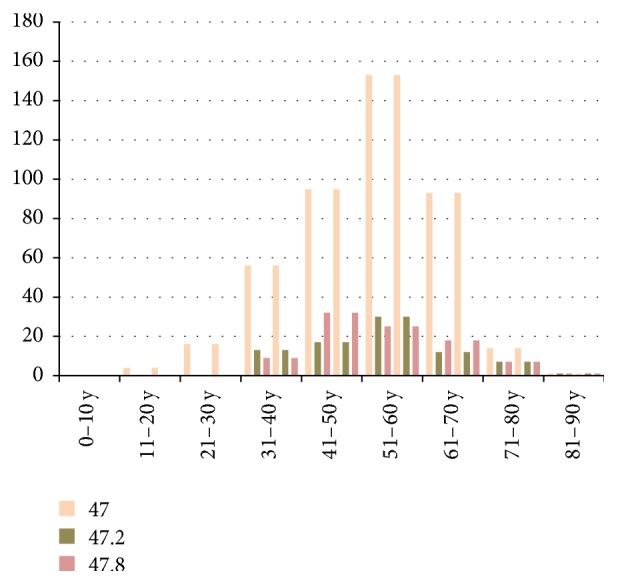
Number of spondylosis MRI scans in ten age ranges, 2011–2015.

Spondylosis was diagnosed in *n* = 608 cases, and more than half of them, *n* = 432 cases, belonged to the general category defined by the code M47. Small number of spondylosis cases were defined by more specific codes (see below and in Table 2), and the most numerous subtypes belonged to the M47.2 code, defining “other spondylosis with radiculopathy,” *n* = 80, and M47.8 code, defining “other spondylosis including cervical spondylosis, lumbosacral spondylosis and thoracic spondylosis,” *n* = 92. Number of M47 cases gradually increased in years 2011–2014, but their slight decrease in 2015 was observed. The cases classified as M47.2 and M47.8 were diagnosed for the first time in 2013, and these two subtypes presented different frequency distribution pattern. M47.2 dramatically increased in 2015 whereas number of M47.8 cases decreased at the same time. The other subtypes constituting also less populous groups appeared for the first time in 2013, and numbers of patients in these subgroups were similar and relatively constant in the following years (*n* = 1–3; Table 2). All analyzed disease subtypes were more often diagnosed in the female (>60%) than in the male subjects (Tables 3 and 4), and most diagnoses of both female and male patients occurred in the age group of 51–60 years (Figure 1).

**Figure 2:**
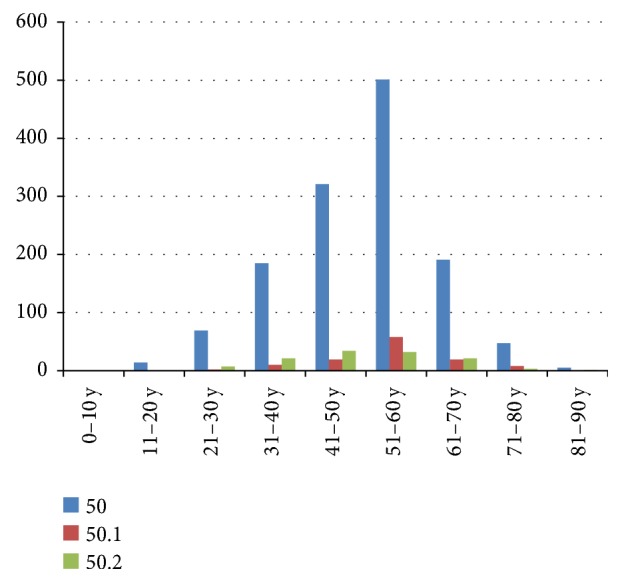
Number of cervical disc disorders MRI scans in ten age ranges, 2011–2015.

Cervical disc disorders were diagnosed in *n* = 1659 cases during the entire study period, and the vast majority of them, *n* = 1344, were defined by the general code M50. Within this group, most numerous subtypes were M50.1, defining cervical disc disorder with radiculopathy represented by *n* = 116 cases, and M50.2 including other cervical disc displacements represented by *n* = 119 cases. M50 cases were gradually increasing in numbers in years 2011–2013 and then started to drop in years 2014-2015. Moreover, cases classified as M50.1 and M50.2 were recorded for the first time in 2013, and these two subtypes had a different frequency distribution patterns: M50.1 cases increased in numbers in years 2013-2014 and then decreased in 2015, whereas in the same time period the number of M50.2 cases was steadily raising. The other and more sporadic disease groups were for the first time reported in 2013, and the number of these rare cases increased in 2014 and was at similar level in 2015 (*n* = 30–40; Table 2). The reports available to us did not contain detailed information about the localization of the neck pain. All analyzed M50 subtypes were more often found in the female than in the male patients, female patients accounting for more than 60% (Tables 3 and 4). In both sexes the most frequently diagnosed patients were in the age group of 51–60 years (Figure 2).

## 4. Discussion

Here we have shown that the cervical disc disorders in the general category M50 were the second most common type of musculoskeletal disorders diagnosed by magnetic resonance imaging tests in the Warmia and Mazury Province in years 2011–2015, following intervertebral disc disorders in the M51 disease category we have described elsewhere [5]. Two subtype codes including M50.1, cervical disc disorder with radiculopathy, and M50.2, other cervical disc displacement, were reported in years 2013–2015. The third group of musculoskeletal disorders diagnosed with the MRI were spondylosis M47 cases, although they were less frequently reported compared to the M51 and M50 disease categories. Within the general M47 category of spondyloses, cases of spondylosis with radiculopathy defined by M47.2 and other spondylosis including cervical spondylosis, lumbosacral spondylosis, and thoracic spondylosis defined by M47.8 codes were also reported. Interestingly, common features of all three general spinal disease groups included diagnoses more frequent in the female (>60%) than in the male subjects and in the age group of 51–60 years.

The neck problems are usually the first symptoms of the cervical disorders and the reason for the detailed radiological examination. The symptoms of neck problems have been very often described by patients such as severe neck pain radiating to the shoulder and upper limb. Neurologic status of those patients was usually characterized by motor and sensory loss in different parts of arm and forearm [14, 26]. Unfortunately, there were no details about the localization of the neck pain in our study population.

Cases of spinal injuries occurring during the road accidents are frequently reported in the literature, and from 30% to 75% of these cases are related to the cervical injures [27–35]. Some researches have shown that during traumatic and nontraumatic accidents the fractures or dislocation of vertebrae or damage of soft tissue including the spinal cord was usually found at C4–C8 level [28, 29, 31, 33, 36, 37] and low intervertebral disc levels were more exposed to damage because of their size [38]. Earliest studies have shown that there was a wide age range of patients with various kinds of cervical injures, but the largest numbers of cases were between 31 and 59 years [27, 28, 31, 33, 34]. Moreover, reported cervical injury cases related mainly to man rather than woman [27, 31, 32, 39].

The cases of cervical radiculopathy are rare and perhaps therefore rarely reported; in our study there was also only a small fraction of a study group. The radiculopathy in Minnesota population prevalence was 107.3 for men and 63.5 for women (per 100,000 population) and it was the highest at the age of 50–54 years [15]. The most common cause of cervical radiculopathy is due to a combination of factors such as decreased disc height and degenerative changes of vertebral column joints and herniation of the nucleus pulposus of intervertebral disc [14, 15, 30].

Some researches have found that cervical vertebra can be injured by frontal or rear impacts during vehicle accidents or during sports and game activities [27–32]. Injuries of cervical vertebra may then result in damage to intervertebral discs including disk displacement and/or compression on the spinal nerve roots [13]. Additionally, it has been shown that each intervertebral level of cervical disc tissue had a different limit for physiological and impact challenge. The C2-C3 middle disc annular tissue was found to be the most sensitive, and it was destroyed at the muscle force replication of 6 g, whereas C3-C4 and C5-C6 discs were damaged at muscle force replication of 10 g [13]. Studies on Minnesota patients population with cervical radiculopathy have shown that the most frequent was monoradiculopathy involving C7 and C6 nerve roots and only in 14% of all cases physical exertion or trauma has occurred [15]. This suggests that in the vertebral cervical section radiculopathy can partly be caused by other factors like spondylosis.

An annual incidence rate of cervical spondylotic radiculopathy varies greatly between populations. For example, in Sicilian population it was 3.5 per 1000 being highest at the age of 50–59 years [40], whereas in the USA 30% and in Ethiopia 9% of all hospitalized patients have shown nontraumatic cervical spondylosis [39, 41].

Most of spondylosis cases were connected with the lumbar part of vertebral column (lumbar spondylosis) and they presented degenerative changes in the lumbar spine [2–4]. In the USA lumbar spondylosis was one of the fastest growing reasons for spinal surgery in adults [42]. The spondylosis surgery is usually performed in either of the two ways: discectomy or chemonucleolysis. The meta-analysis data of surgical cases have demonstrated that patients had better clinical outcomes following discectomy than after chemonucleolysis, and discectomy was much more effective in treating of sciatica patients [4]. According to the same meta-analysis data, in the 80s and 90s of 20th century spondylosis occurred more often in man than woman regardless of the age ranges [4]. Nowadays, the same situation was observed in the Japanese population, where the spondylosis was more frequent in man than in woman, and the age was the strongest among many factors associated with the disease [3, 43].

The group of spondyloses contains also arthrosis or osteoarthritis of spine degeneration of facet joints. Some studies have shown that MRI can precisely visualize facet joint osteoarthritis [42–44]. Studies on the cervical and lumbar facet joins have shown that the thickness and width of joint cartilage depended on the region of spine, sex, and location [42–44]. Moreover, changes in tissue structure due to osteoarthritis are thought to be strongly connected with disc degeneration, and in adult facet joint osteoarthritis, the disease follows the onset of disc degeneration [43]. 

*Conclusion.* Our study shows that while the largest fraction of patients suffering from any of the spine disorders is in the 51–60-year age group, a significant number of patients first present with these diseases at much younger age of 31–40 years in the Warmia and Mazury Province. This observation points to an urgency of developing and introducing methods preventing disorders of the vertebral column at a younger age, preferably at a school age. Additionally, this study also demonstrates the significance of access to MRI as a method of choice in early and reliable diagnosis of pathological changes in the spinal column.

## Figures and Tables

**Table 1:** Percentage of M category diseases among all spinal diseases.

Percentage of disease codes [%]	2011	2012	2013	2014	2015	2011–2015
M40	0	0,00	0,00	0,13	0,07	0,05
M41	0,14	0,04	0,17	0,16	0,18	0,14
M42	0,16	0,04	0,03	0,03	0,00	0,03
M43	0,07	0,09	0,03	0,03	0,04	0,05
M45	0,00	0,00	0,00	0,06	0,11	0,04
M46	0,00	0,04	0,03	0,00	0,00	0,02
M47	3,81	3,29	3,74	5,38	6,14	**4,57**
M48	0,00	0,00	0,48	1,34	0,77	0,61
M49	0,14	0,00	0,00	0,00	0,00	0,02
M50	10,88	11,11	13,58	14,40	10,92	**12,48**
M51	28,90	38,85	25,72	30,33	31,73	**30,75**

**Table 2:** Number of M47 and M50 categories diseases.

Types of category M diseases	2011	2012	2013	2014	2015	2011–2015
M47						
M47	55	77	85	112	103	432
M47.2	0	0	4	11	65	80
M47.8	0	0	40	46	6	92
Others of M47	0	0	3	3	1	7

Total of M47	55	77	132	172	176	611

M50						
M50	157	260	448	322	157	1344
M50.1	0	0	23	55	38	116
M50.2	0	0	6	40	73	119
Others of M50	0	0	2	40	38	80

Total of M50	157	260	479	452	311	1659

**Table 3:** Number of M47 and M50 categories diseases in each year for female (F) and male (M).

Code desease	2011		2012		2013		2014		2015		2011–2015
	F	M	F	M	F	M	F	M	F	M	
M47	37	18	46	31	55	30	64	48	63	40	432
M47.0	0	0	0	0	0	3		0	0	0	3
M47.2	0	0	0	0	4	0	8	3	50	15	80
M47.8	0	0	0	0	29	11	33	13	4	2	92
M47.9	0	0	0	0	0	0	2	1	1	0	4
M50	104	53	184	76	303	145	227	95	102	55	1344
M50.0	0	0	0	0	1	1	5	6	3	0	16
M50.1	0	0	0	0	15	7	36	19	25	14	116
M50.2	0	0	0	0	5	1	25	19	45	24	119
M50.3	0	0	0	0	1	0	8	9	26	10	54
M50.8	0	0	0	0	0	0	5	1	3	1	10

**Table 4:** The pattern of percentage of female (F) and male (M) in analyzed diseases in the most numerous subtypes of patients of 51–60-year age range.

	2011		2012		2013		2014		2015	
	F	M	F	M	F	M	F	M	F	M
M47	69,09	30,91	61,04	38,96	65,88	34,12	58,04	41,96	61,17	38,83
M47.2	0	0	0	0	75,00	25,00	72,73	27,27	46,15	53,85
M47.8	0	0	0	0	75,00	25,00	71,74	28,26	66,67	33,33
M50	67,52	32.48	69,62	30,38	67,86	32,14	71,12	28,88	68,79	31,21
M50.1	0	0	0	0	65,22	34,78	65,45	34,55	63,16	36,84
M50.2	0	0	0	0	83,33	11,67	65,00	35,00	63,01	36,99
